# Copigmentation with Chlorogenic and Ferulic Acid Affected Color and Anthocyanin Stability in Model Beverages Colored with *Sambucus peruviana*, *Sambucus nigra*, and *Daucus carota* during Storage

**DOI:** 10.3390/foods9101476

**Published:** 2020-10-16

**Authors:** Nuryati P. Pangestu, Gonzalo Miyagusuku-Cruzado, M. Monica Giusti

**Affiliations:** Department of Food Science and Technology, The Ohio State University, 2015 Fyffe Rd, Columbus, OH 43210-1007, USA; nuryati.pangestu@gmail.com (N.P.P.); miyagusukucruzado.1@osu.edu (G.M.-C.)

**Keywords:** copigmentation, anthocyanins, pyranoanthocyanins, color stability

## Abstract

The food industry is looking for alternatives to synthetic colorants. Anthocyanins (ACNs) are suitable replacements due to their color characteristics and potential health benefits. The application of sauco (*Sambucus peruviana*, SP) as a potential source of ACN-based colorants was evaluated and compared to elderberry (*Sambucus nigra*, SN) and an extract from purple carrots (PC). Color and pigment stability were evaluated using a model beverage system during eight weeks of storage. Copigmentation with chlorogenic acid (CGA) and ferulic acid (FA) were also evaluated. SP ACNs provided darker and more intense colors than those obtained with SN but less intense than those obtained with PC. Addition of CGA and FA resulted in significantly darker colors with higher chroma in beverages colored with SP and SN but not in beverages colored with PC. Copigmentation with FA reduced monomeric pigment half-lives for all ACN sources but increased the chroma half-lives of beverages colored with SP and SN, from 23 to 49 weeks, and from 23 to 55 weeks, respectively. Analyses using liquid chromatography coupled to photodiode array detection and mass spectrometry showed that interaction between non-acylated ACNs and FA resulted in the formation of pyranoanthocyanins. Overall, ACNs from non-acylated sources such as SP, in combination with FA, showed potential for commercial food applications.

## 1. Introduction

Color is defined as a sensation perceived by an individual when light within the visible spectrum falls upon the retina. It plays a major role in the acceptability of foods, as consumers tend to use color as an indicator of flavor, safety, texture, and nutritional value [1]. For centuries, the food industry has used colorants to ensure uniformity or improve the original appearance of foods [2]. Interest and demand for colorants from natural sources have increased considerably because of legislative action and consumer concerns over the use of artificial additives in foods [1,2,3]. Some undesirable effects of synthetic food colorants are related to allergy, hypersensitivity, intolerance, and hyperactivity in children [3,4,5].

Anthocyanins (ACNs) are water-soluble pigments responsible for imparting attractive colors in plant tissues, including fruits, flowers and grains [6]. Additionally, ACN consumption has shown potential health benefits such as free radical scavenging and anti-inflammatory activity [7], lessening capillary permeability and fragility [8], obesity prevention [9], as well as anti-neoplasic and anti-tumor properties [10]. Therefore, the application of ACN-rich extracts as food colorants may provide both attractive colors and nutritional benefits.

*Sambucus peruviana* (SP) is a Peruvian elderberry, which has a pleasant flavor and is commonly known as sauco. SP ACN profile is composed by only non-acylated ACNs [11,12]. *Sambucus nigra* (SN) is an European elderberry containing non-acylated ACNs [13], whose pigments have been widely used as colorants for various foods and beverages such as juices, wines, jams, and canned fruits [14,15,16,17]. Typically, non-acylated ACNs show limited stability in food matrices [2]. However, addition of copigments can enhance pigment stability during storage [18], spray drying [19], thermal processing [20], and high-pressure processing [21]. Moreover, studies have shown that copigmentation with phenolic acids resulted in an improvement of color and color stability of ACN-rich extracts from fruits and berries in a wide variety of food matrices such as juices, purees, jams, and syrups [22,23,24,25]. *Daucus carota* or purple carrot (PC), contains ACNs with the same anthocyanidin as SN and SP; however, its major ACNs are acylated with hydroxycinnamic acids [26]. It has been reported that acylated ACNs have a higher stability during storage than their non-acylated counterparts [2].

Ferulic acid (FA) is a ubiquitous hydroxycinnamic acid that can be found in the seeds and leaves of a wide variety of food plants with multiple applications in the food industry such as a food preservative and a color protecting agent [27]. Moreover FA and compounds bearing a feruloyl moiety were shown to have anti-inflammatory, antidiabetic, and anticancer activity [27,28]. Chlorogenic acid (CGA) is one of the most available phenolic acids that can be found in abundance in coffee, tea, citrus fruits, and berries [29]. CGA can be used as a food additive due to its antimicrobial and antioxidant properties, and its ability to inhibit the degradation of other phytochemicals such as ACNs [30]. Moreover, CGA has been shown to have antidiabetic, anti-obesity, antioxidant, anti-inflammatory, and anti-hypertension effects upon consumption [29,30]. Therefore, due to their wide availability, many plant extracts can be used as natural sources for FA and CGA without further purification, acting not only as color stabilizing agents but also as bioactive natural extracts.

The prolonged interaction of ACNs with some phenolic compounds such as hydroxycinnamic acids can result in the formation of more stable ACN-derived pigments called pyranoanthocyanins (PACNs) [26]. These ACN-derived pigments are characterized by the presence of an extra pyran ring formed through the cycloaddition of a reactive adduct onto position C-4 and the 5-OH group of the ACN [31]. PACN formation has not been reported to occur spontaneously in solutions containing acylated ACNs, thus requiring the addition of an exogenous cofactor to promote their formation, despite having a hydroxycinnamic acid present in the ACN structure [26]. Generally, these compounds have a different UV-Vis spectrum than their respective ACN predecessor along with an improved resistance to bleaching and enhanced stability during storage [32]. PACNs formed with hydroxycinnamic acids have stronger antioxidant and similar anti-inflammatory activities than their ACN counterparts [33,34]. These bioactive characteristics combined with their higher resistance to degradation under gastrointestinal conditions [35] may result in an enhanced bio-effectiveness and bioactivity of PACNs.

In this study, we evaluated the application of SP as a new source of ACN-based food colorant, using a beverage model system. The changes in color and pigment stability were measured during an eight-week storage period and compared model beverages colored with commercial extracts from SN and PC. Moreover, the effects on color and pigment stability of two types of copigment (CGA or FA) were also evaluated, as well as the possible formation of PACNs during storage.

## 2. Materials and Methods

### 2.1. Materials

#### 2.1.1. Chemicals

Isosweet 100 high-fructose corn syrup was obtained from Tate & Lyle (Decatur, IL, USA), potassium sorbate from Spectrum (Gardena, CA, USA), sodium benzoate from Alfa Aesar (Heysham, Lancaster, UK), and sodium bicarbonate from Sigma Chemical Co. (St. Louis, MO, USA). Citric acid, mass spectrometry (MS) grade water, acetonitrile, methanol, formic acid, hydrochloric acid, and high pressure liquid chromatography (HPLC) grade acetone were purchased from Fisher Scientific (Fair Lawn, NJ, USA). Chloroform and ethyl acetate were obtained from Mallinckrodt Chemicals (Phillipsburg, NJ, USA). CGA and FA were purchased from MP Biochemicals, LLC (Solon, OH, USA). All reagents were of analytical grade unless otherwise indicated.

#### 2.1.2. Plant Materials and ACN Extracts

SP berries were harvested in Andahuaylas, Apurimac, Peru. Berries were washed, blended, and freeze-dried in a 4.5 L capacity Labconco freeze dryer (Kansas City, MO, USA) at −48 ± 2 °C at the laboratories of the Universidad Nacional Agraria La Molina in Lima, Peru. Dried samples were refrigerated at 4 °C and sent to the Ohio State University in Columbus, OH, USA for further analyses. SN extract was donated by Artemis International Inc. (Fort Wayne, IN, USA), and PC extract was donated by Diana Vegetal (Antrain, France).

### 2.2. Methods

#### 2.2.1. ACN Extraction from SP

ACNs were extracted following the methodology described by Rodriguez-Saona and Wrolstad [36]. Briefly, freeze-dried SP powdered berries were extracted by dispersing 20 g of powder with 50 mL of 0.01% HCl acidified water (*v*/*v*) overnight, and then blending the reconstituted powder with 50 mL of acetone for 3 min using a Waring blender. The slurry was then passed through a Whatman No. 4 filter paper using a Büchner Funnel. The cake was re-extracted using 70% aqueous acetone (*v*/*v*) until all pigments had been extracted from the cake. The ACN-rich solution was then placed into a separatory funnel and partitioned with 2 volumes of chloroform. Solutions were gently mixed and let stand overnight at 4 °C to ensure adequate separation. The ACN-rich aqueous top layer was collected, and residual acetone/chloroform was evaporated using a Büchi rotary evaporator (Büchi Laboratories, Postfach, Switzerland) at 40 °C under vacuum. The solution was then brought to 100 mL in a volumetric flask using 0.01% HCl acidified water (*v*/*v*).

#### 2.2.2. ACN Semi-purification

ACN semi-purification was performed according to the method described by Rodriguez-Saona and Wrolstad [36]. Briefly, samples were passed through a C_18_ Sep-Pak cartridge (6 cc, 1 g, Waters Corp, Milford, MA, USA), previously activated with methanol and 0.01% HCl acidified water (*v*/*v*). ACNs and other phenolics were adsorbed onto the cartridge, while sugars, acids and water-soluble compounds were eluted with two volumes of acidified water. Ethyl acetate was used to remove other phenolic compounds such as phenolic acids and flavonols. The adsorbed ACNs were then eluted with 0.01% HCl acidified methanol (*v*/*v*). The ACN-rich methanolic extract was then concentrated using a rotary evaporator at 40 °C, and the pigments were redissolved in acidified water.

#### 2.2.3. Monomeric ACN and Molymeric Color Quantification

The monomeric ACN content in ACN-rich extracts and model beverages was determined using the pH differential method, and the percent of polymeric color was determined using indices for pigment degradation, color density and polymeric color [37]. A Shimadzu 2450 spectrophotometer (Shimadzu, Columbia, MD, USA) was used for spectral measurements at 420, 520 and 700 nm with 1 cm pathlength disposable cuvettes. Pigment content was calculated as cyanidin-3-glucoside equivalents (cy-3-glu eq.), using a molecular weight of 449.3 g/mol and an extinction coefficient of 26,900 L mol^−1^ cm^−1^.

#### 2.2.4. Preparation of Model Beverage Systems

Model beverages were prepared according to the method described by Rodriguez-Saona et al. [38]. Briefly, a 10° Brix solution containing high fructose corn syrup, 0.1% potassium sorbate (*w*/*v*), 0.1% sodium benzoate (*w*/*v*), and 0.1 M citric acid was prepared. The pH of the solution was adjusted to 3.5 with sodium bicarbonate when needed. ACNs were added to model beverages for a final concentration of 20 mg cy-3glu eq./100 mL model beverage. CGA or FA were added as copigments in an ACN:copigment molar proportion of 1:10 as suggested by Rein and Heinonen [24].

#### 2.2.5. Storage Studies

Ten milliliters of model beverages colored with ACNs with and without copigments were placed in glass vials, flushed with nitrogen, capped, and pasteurized in water at 85 °C for 25 min. Vials were stored in a 25 °C incubator for eight weeks; during which, monomeric ACN content, percent of polymeric color, as well as color parameters were monitored. Measurements were conducted in triplicate every two weeks (0, 2, 4, 6 and 8 weeks).

#### 2.2.6. HPLC Coupled with Photodiode Array Detection and Electrospray MS (HPLC-PDA-ESI-MS) Analyses of Model Beverages Colored with ACNs

The ACN profile of model beverages was analyzed at 0, 4, and 8 weeks using an HPLC system equipped with two LC-20AD pumps, a SIL-20AC autosampler and an SPD-M20A photodiode array (PDA) detector coupled to a LCMS-2010 mass spectrometer (Shimadzu, Columbia, MD, USA). Briefly, 50 µL of model beverage colored with SP or SN were injected to a reversed-phase C_18_ Allsphere ODS-2 column (5 µm, 4.6 × 250 mm; Grace Davison Discovery Sciences, Deerfield, MA, USA) with a Symmetry 2 micro guard column (4.6 × 22 mm; Waters Corp, MA, USA). Separation was achieved using a binary solvent system of 5% (*v*/*v*) formic acid in water (solvent A) and 100% acetonitrile (solvent B). A linear gradient from 5–15% B at 8 min, 15–20% B at 15 min, 20–50% B at 30 min, 50% B for 3 min and 50–5% B at 37 min, was used at a flow rate of 1 mL/min. Due to the acylated nature of PC ACNs, a different column and gradient were used for chromatographic separation. Briefly, 50 µL of model beverage colored with PC ACNs were injected to a reversed-phase Symmetry C_18_ column (3.5 µm, 4.6 × 150 mm; Waters Corp., Milford, MA, USA) with a Symmetry 2 micro guard column (4.6 × 22 mm; Waters Corp., Milford, MA, USA). Separation was achieved using a linear gradient from 5–20% B at 18 min, 20% B at 22 min, 20–5% B at 25 min, and 5% B at 30 min at a flow rate of 0.8 mL/min.

A flow rate of 0.2 mL/min was diverted to the mass spectrometer. Mass spectrometry analyses were conducted on a single quadrupole ion-tunnel mass spectrometer equipped with an electrospray ionization interface (ESI). Analyses were performed under positive ion mode with the following settings: nebulizing gas flow, 1.5 L/min; interface bias, +4.5 kV; heat block temperature, 200 °C; focus lens, −2.5 V; entrance lens, −50 V; pre-rod bias, −3.6 V; main-rod bias, −3.5 V; detector voltage, 1.5 kV; scan speed, 2000 amu/sec. A full scan (total ion count, TIC) was performed with a mass range from 250–1200 *m*/*z* and selected ion monitoring (SIM) was used to look for the molecular ions of the six most common ACN aglycones during the analysis (*m*/*z* 271, 287, 301, 303, 317, 331). LCMS Solutions software (Version 3.0, Shimadzu, Columbia, MD, USA) was used to analyze and graphically represent the HPLC-PDA-ESI-MS data.

#### 2.2.7. Color Measurements

An aliquot of each model beverage (≈2 mL) was transferred to a 2 mm pathlength precision cell made of optical glass (Hellma, Plainview, NY, USA) and read for CIELab parameters, lightness (L*), chroma (C*) and hue angle (h*) using a Hunter Colorquest XE (HunterLab, Hunter Associates Laboratories Inc., Reston, VA, USA). The equipment was set for total transmittance, D65 illuminant, and a 10° observer angle.

#### 2.2.8. Statistical Analyses

The results from color measurements, monomeric ACN quantification, and percent of polymeric color were subjected to regression analyses and one-way analysis of variance (ANOVA). Chroma and monomeric ACN concentration were plotted against time, and linear regression analyses were used to determine the adequacy of the degradation kinetic model. All statistical analyses were performed using SPSS 15.0 software (SPSS Inc., Chicago, IL, USA), and *p* < 0.05 was considered statistically significant. All experiments were conducted in triplicate.

## 3. Results and Discussion

To assess the feasibility of using SP as a potential new source of ACN-based food colorants, the color characteristics and pigment stability of a model beverage colored with a semi-purified ACN extract from SP were evaluated over an eight-week storage period. These results were compared to a commercially used SN extract and a commercially available ACN extract from PC containing acylated ACNs.

### 3.1. Initial Color Characteristics of Model Beverages

The initial color characteristics of all model beverages are shown in Table 1. Non-acylated ACNs from SP provided a bright intense red color (L* = 56.79 ± 0.04, chroma = 59.51 ± 0.10, hue angle = 15.53 ± 0.07°) that were darker, more intense, and similar in hue to those obtained with the extract from SN (L* = 69.13 ± 0.17, chroma = 47.18 ± 0.27, hue angle = 11.08 ± 0.06°). Model beverages colored with PC extract displayed a darker orange/red color at a higher hue angle with more color intensity (L* = 44.11 ± 0.03, chroma = 71.34 ± 0.01, hue angle = 24.78 ± 0.03°) than the ones colored with SP. The effect of acylation on chroma values was significant (*p* < 0.05), as model beverages colored with acylated ACNs from PC had a higher chroma than those colored with extracts containing only non-acylated ACNs (SP and SN). Addition of copigments (CGA and FA) resulted in more intense colors with significantly higher chroma values (*p* < 0.05), and less yellow tones denoted by significantly lower hue angles (*p* < 0.05). The color of model beverages with non-acylated ACNs from SP and SN displayed a significantly darker color (*p* < 0.05) after copigment addition. However, the presence of copigments did not result in significant changes in the lightness (L*) of model beverages colored with PC extract. Similar color enhancements have been reported for ACNs copigmented with phenolics, resulting in hyperchromic effects and color improvements due to a higher π-electron delocalization [39,40]. When comparing copigments, addition of CGA resulted in a more intense color than model beverages with FA. This was denoted by a significantly higher chroma for model beverages colored with SN (*p* < 0.05) and PC (*p* < 0.05) and copigmented with CGA. However, no significant differences in the chroma of model beverages colored with SP ACNs were observed when copigmented with either CGA or FA. Moreover, when compared against model beverages copigmented with FA, CGA addition resulted in a significantly higher hue angle for all colorant sources (*p* < 0.05).

### 3.2. Color Stability and Monomeric ACN Content in Model Beverages

Results in Table 2 showed that for all colorant sources in this study, there was a significant decrease in chroma and monomeric ACN content over time (*p* < 0.01), despite the presence of copigments. It has been widely reported that colorants from natural sources fade away or lose color during storage, regardless of the type of pretreatment they were subjected to [17,41,42]. In model beverages without copigments, results showed that after eight weeks, the chroma for beverages colored with PC was significantly higher than that observed for SP and SN (*p* < 0.01, data not shown). Regression analyses for model beverages colored with SP, SN or PC ACNs, showed that color degradation and monomeric ACN decrease followed a zero-order degradation kinetic model. Moreover, results showed that this behavior was not affected by the presence of a copigment. This is consistent with previous studies reporting similar degradation models for acylated ACNs from purple sweet potato [43], purple corn [44], and red radish [45]. Literature has shown that ACN acylation improves the color and stability of the pigment, mostly due to intramolecular copigmentation, with the anthocyanidin acting as a chromophore and the acyl group as electron-donor copigments [2,46,47]. However, results seem to indicate that acylation did not seem to modify the degradation model, but only the degradation speed.

Results in Table 2 also showed that, in the absence of copigments, the half-life of the chroma of model beverages colored with SP and SN was similar (23 weeks), but shorter than the half-life of beverages colored with acylated ACNs form PC (26 weeks). Copigmentation of non-acylated ACNs with FA significantly decreased the degradation rate of the chroma (*p* < 0.01), thus extending their half-life from 23 to 49 weeks for SP, and from 23 to 55 weeks for SN. However, this effect was not observed in model beverages colored with PC, where addition of FA resulted in a decreased half-life of the chroma from 26 to 19 weeks. This behavior could be explained by previous literature that has shown that copigmentation of acylated ACNs with hydroxycinnamic acids resulted in a lower color stability, as it was hypothesized that the protective intramolecular mechanism of acylated ACNs was disrupted by exogenous ferulic and caffeic acid [39,48]. Nonetheless, this disrupting effect was not found for other types of phenolic acids such as gallic acid [39], and in this study it seems to be the case for CGA. Interestingly, results in Table 2 showed that in model beverages colored with SP or SN, the increased chroma stability showed by beverages copigmented with FA did not correlate with the shorter half-life of their monomeric ACN content.

### 3.3. Polymeric ACN Content

**Figure 1:**
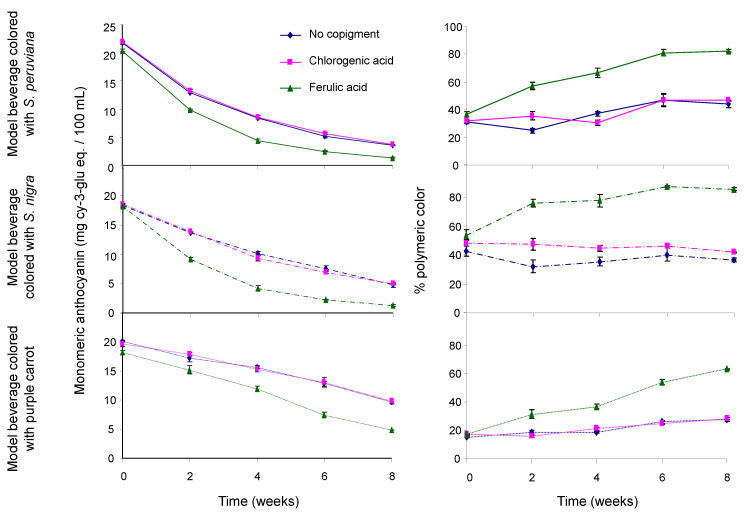
Effect of copigmentation on the monomeric anthocyanin content (left) and percent of polymeric color (right) in model beverages colored with *S. peruviana* (upper), *S. nigra* (middle), and purple carrot (lower). Model beverages with no copigment (blue line), with chlorogenic acid (pink line), and with ferulic acid (green line).

Results in Figure 1 showed a decrease in the monomeric ACN content in model beverages along with an increasing content of polymeric pigments over time. The effect of acylation on polymeric ACN content was significant, as model beverages colored with the PC extract showed a lower polymeric ACN content than those colored with non-acylated pigments (*p* < 0.05). On average over time, there was no significant difference in the polymeric ACN content between model beverages colored with SP and those colored with SN (*p* > 0.05). Results showed that copigmentation with FA resulted in a significantly faster degradation of monomeric ACNs (*p* < 0.05) and a higher polymeric ACN content (*p* < 0.01). Unlike with FA, no significant differences in polymeric pigment content were observed between model beverages copigmented with CGA and those without copigment (*p* > 0.05). Studies have shown that monomeric ACN degradation usually resulted in the formation of polymeric pigments [25,49,50]. However, they also reported that this monomeric ACN degradation should result in a significant decrease in color intensity (chroma), which is not the case of model beverages colored with non-acylated ACNs copigmented with FA. Taken together, these results seem to indicate the presence of an ACN-derived pigment that will form over time with FA, that will not lose color at pH 4.5 when using the pH differential method for quantification of monomeric ACNs, and that will not bleach easily with sodium bisulfite.

### 3.4. HPLC–PDA–ESI–MS Analysis of Pigments in Model Beverages

**Figure 2:**
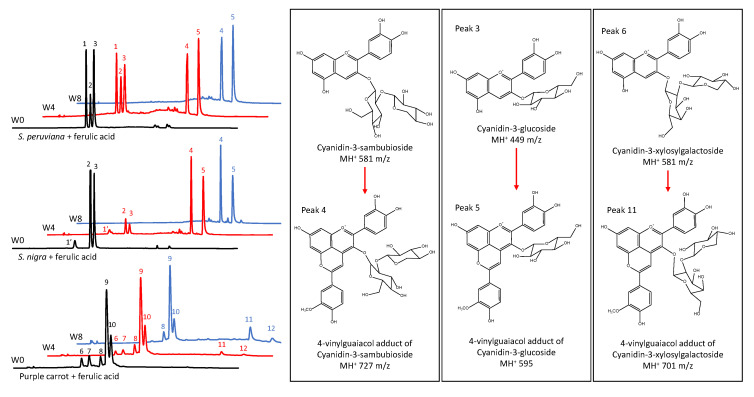
Anthocyanin high pressure liquid chromatography coupled with photodiode array detection (HPLC-PDA) profile of model beverages colored with anthocyanins and copigmented with ferulic acid at week 0 (W0, black line), week 4 (W4, red line) and week 8 (W8, blue line). Model beverages colored with *S. peruviana* (upper), *S. nigra* (middle), and purple carrot (lower).

To determine the ACN profile of model beverages and changes in its composition over time, HPLC–PDA–ESI–MS analyses were conducted at the start of the experiment, and after four, and eight weeks of storage. Results in Figure 2 and Table 3 showed the presence of three major ACNs in model beverages colored with SP, cyanidin-3-lathyroside (peak 1), cyanidin-3-sambubioside (peak 2), and cyanidin-3-glucoside (peak 3). This is consistent with two previous reports on the composition of SP ACN profile [11,12]. In the case of model beverages colored with SN, three major ACNs were found, cyanidin-3-sambubioside-5-glucoside (peak 1′), cyanidin-3-sambubioside (peak 2), and cyanidin-3-glucoside (peak 3). These results are similar to one previously reported for SN [51].

Results showed that the presence of a copigment did not affect the proportion of these ACNs during storage. However, in model beverages colored with SP and SN, the presence of FA as a copigment resulted in the formation of two new peaks (peak 4 and peak 5 in Figure 2) characterized by longer retention times. These shifts in the retention times are remarkably similar to the ones shown for pyruvic acid adducts of ACNs from SN [51]. Mass spectrometry results in Table 3 showed that peaks 4 and 5 are consistent with pyranoanthocyanins of *m*/*z* of 727 and 595, respectively. A *m*/*z* of 727 is consistent with a 4-vinylguaiacol adduct of cyanidin-3-sambubioside or cyanidin-3-lathyroside. These two peaks may have coeluted and, due to their equal *m*/*z* and similar PDA absorption spectra, separation was not possible under our experimental conditions. Additionally, a *m*/*z* of 595 is consistent with a 4-vinylguaiacol adduct of cyanidin-3-glucoside. Similar compounds have been previously reported and denominated pinotins or hydroxyphenyl-pyranoanthocyanins [26,47,52]. Results in Figure 2 also showed that five major ACNs were found in model beverages colored with PC extract. As shown in Table 3, these were identified as cyanidin-3-xylosyl-galactoside (peak 6), cyanidin-3-xylosyl-glucosyl-galactoside (peak 7), cyanidin-3-xylosyl-glucosyl-galactoside acylated with *p*-coumaric acid (peak 8), cyanidin-3-xylosyl-glucosyl-galactoside acylated with FA (peak 9), and cyanidin-3-xylosyl-glucosyl-galactoside acylated with sinapic acid (peak 10). Unlike with non-acylated ACNs from SP and SN, copigmentation of PC ACNs with FA did not result in an enhancement of color intensity (chroma) or stability. However, after eight weeks of storage, two small new peaks were detected by the PDA after chromatographic separation. These may also be pyranoanthocyanins formed from the two minor non-acylated ACNs initially present in the PC extract. Similar results were reported by Schwarz et al. [26], where pyranoanthocyanins from PC were observed after 3 months of storage. As shown in Table 3, peak 11 was tentatively identified as the 4-vinylguaiacol adduct of cyanidin-3-xylosyl-galactoside. However, the *m/z* of peak 12 was not elucidated by the MS, but it was tentatively assigned as a 4-vinylguaiacol adduct of cyanidin-3-xylosyl-glucosyl-galctoside. Moreover, acylated ACNs from PC apparently were not capable of forming pyranoanthocyanins during the duration of this study. Overall, these results showed that prolonged ACN interaction with FA resulted in the formation of pyranoanthocyanins. These ACN-derived pigments have a strong color at pH 1 and do not lose color at pH 4.5 [32], which in our study resulted in an underestimation of the monomeric pigment content when using the pH differential method. Moreover, these pigments are resistant to bisulfite bleaching due to the unavailability of position C4 of the original ACN structure, preventing the nucleophilic attack of bisulfite [32], which in our study resulted in an overestimation of the polymeric content. The presence of pyranoanthocyanins and their formation over time may explain the changes in the color characteristics as wells as in monomeric and polymeric ACN content in model beverages colored with ACNs and copigmented with FA.

## 4. Conclusions

SP ACNs provided color characteristics and stability to model beverages, comparable to a commercially available ACN extract from SN. However, its color characteristics were not as strong as in beverages colored with a PC extract rich in acylated ACNs. Addition of CGA as a copigment improved the initial color of model beverages colored with SP ACNs. However, it did not affect beverage color or pigment stability during storage. Interestingly, copigmentation with FA resulted in a more stable color but with lower monomeric ACN content and higher polymeric pigment content, due to the formation of pyranoanthocyanins. Overall, ACNs from SP, in combination with FA, showed potential to be used for commercial food and beverage applications as a natural and appealing value-added product. Future studies will focus on the efficient production of hydroxyphenyl-pyranoanthocyanins and their potential use as food colorants in different food matrices.

## Figures and Tables

**Table 1:** Initial color characteristics of model beverages colored with *S. peruviana*, *S. nigra*, and purple carrot extract with and without chlorogenic and ferulic acids as copigments.

Colorant	Copigment	L*	Chroma	Hue Angle
*S. peruviana*	No copigment	56.79 ± 0.04 ^a^	59.51 ± 0.10 ^a^	15.53 ± 0.07 ^a^
	Chlorogenic acid	54.46 ± 0.35 ^b^	62.15 ± 0.22 ^b^	13.85 ± 0.10 ^b^
	Ferulic acid	54.70 ± 0.53 ^b^	61.53 ± 0.54 ^b^	12.52 ± 0.33 ^c^
*S. nigra*	No copigment	69.13 ± 0.17 ^a^	47.18 ± 0.27 ^a^	11.08 ± 0.06 ^a^
	Chlorogenic acid	65.16 ± 0.11 ^b^	52.66 ± 0.14 ^b^	7.95 ± 0.08 ^b^
	Ferulic acid	65.87 ± 0.55 ^b^	50.54 ± 0.80 ^c^	5.78 ± 0.60 ^c^
Purple carrot extract	No copigment	44.11 ± 0.03 ^a^	71.34 ± 0.01 ^a^	24.78 ± 0.03 ^a^
	Chlorogenic acid	43.79 ± 0.18 ^a^	70.38 ± 0.18 ^b^	22.84 ± 0.20 ^b^
	Ferulic acid	43.69 ± 0.12 ^a^	69.44 ± 0.08 ^c^	21.48 ± 0.17 ^c^

Different letters indicate significant differences against model beverages without copigments for the same anthocyanin source.

**Table 2:** Degradation models and half-lives of color chroma and monomeric anthocyanins in model beverages with and without copigments.

Colorant	Copigment	Chroma			Monomeric Anthocyanins		
		Equation	Half-Life (weeks)	*R* ^2^	Equation	Half-Life (days)	*R* ^2^
*S. peruviana*	No copigment	*y* = −0.039*x* + 1.775	23	1.00	*y* = −0.098*x* + 1.320	47	0.99
	Chlorogenic acid	*y* = −0.039*x* + 1.802	23	1.00	*y* = −0.097*x* + 1.327	47	1.00
	Ferulic acid	*y* = −0.018*x* + 1.769	49	0.90	*y* = −0.071*x* + 1.279	30	0.99
*S. nigra*	No copigment	*y* = −0.037*x* + 1.685	23	0.97	*y* = −0.071*x* + 1.279	63	0.98
	Chlorogenic acid	*y* = −0.041*x* + 1.739	21	0.98	*y* = −0.072*x* + 1.274	62	0.99
	Ferulic acid	*y* = −0.015*x* + 1.683	55	0.85	*y* = −0.146*x* + 1.245	29	0.99
Purple carrot extract	No copigment	*y* = −1.414*x* + 72.213	26	0.92	*y* = −1.257*x* + 20.154	56	0.97
	Chlorogenic acid	*y* = −1.626*x* + 71.143	22	0.89	*y* = −1.237*x* + 20.125	58	0.95
	Ferulic acid	*y* = −1.865*x* + 71.035	19	0.90	*y* = −1.723*x* + 18.451	38	0.98

**Table 3:** HPLC-PDA with electrospray mass spectrometry characterization and peak area percentage of anthocyanin (ACN) and anthocyanin-derived pigments in model beverages colored with *S. peruviana*, *S. nigra*, and purple carrot before (W0) and after eight weeks of storage (W8). CGA: chlorogenic acid, FA: ferulic acid.

Peak	ID	*m/z*	ACN Only		CGA		FA	
			W0	W8	W0	W8	W0	W8
In model beverages colored with *S. peruviana*								
1	Cyanidin-3-lathyroside	581,287	32	37	34	37	34	0
2	Cyanidin-3-sambubioside	581,287	23	26	21	26	17	0
3	Cyanidin-3-glucoside	449,287	45	37	45	36	46	0
4	Vinylguaiacol adduct of cyanidin-3-sambubioside and vinylguaiacol adduct of cyanidin-3-lathyroside	727	-	-	-	-	2	45
5	Vinylguaiacol adduct of cyanidin-3-glucoside	595	-	-	-	-	2	54
In model beverages colored with *S. nigra*								
1′	Cyanidin-3-sambubioside-5-glucoside	744,287	6	14	8	28	7	0
2	Cyanidin-3-sambubioside	581,287	50	36	58	40	52	0
3	Cyanidin-3-glucoside	449,287	44	31	34	32	38	0
4	Vinylguaiacol adduct of cyanidin-3-sambubioside	727	-	-	-	-	1	50
5	Vinylguaiacol adduct of cyanidin-3-glucoside	595	-	-	-	-	1	50
In model beverages colored with purple carrot								
6	Cyanidin-3-xylosyl-galactoside	581,287	6	3	6	3	5	1
7	Cyanidin-3-xylosyl-glucosyl-galactoside	744,287	8	2	8	2	7	1
8	Cyanidin-3-xylosyl-glucosyl-galactoside acylated with p-coumaric acid	889,287	5	5	5	5	5	6
9	Cyanidin-3-xylosyl-glucosyl-galactoside acylated with ferulic acid	919,287	56	64	56	66	57	54
10	Cyanidin-3-xylosyl-glucosyl-galactoside acylated with sinapic acid	949,287	25	26	25	24	25	18
11	Vinylguaiacol adduct of cyanidin-3-xylosyl-galactoside	701	-	-	-	-	0	13
12	Vinylguaiacol adduct of cyanidin-3-xylosyl-glucosyl-galactoside *	ND	-	-	-	-	0	7

ND: Not detected, asterisk (*) denotes tentative identification.
